# Changes in adiposity indices over 10 years and risk of type 2 diabetes: The Whitehall II cohort study

**DOI:** 10.1111/dom.16615

**Published:** 2025-07-14

**Authors:** Cunrong Huang, Yuntao Chen, Annie Britton

**Affiliations:** ^1^ Research Department of Epidemiology and Public Health University College London London UK; ^2^ Faculty of Brain Science University College London London UK

**Keywords:** adiposity index, change rate, obesity, type 2 diabetes, waist circumference

## Abstract

**Aims:**

Common adiposity indices that are used to assess obesity include body mass index (BMI), waist circumference (WC) and waist‐to‐height ratio (WHtR). We aim to investigate change rates of the three indices in British middle‐aged adults and compare effects of different adiposity indices' changes on incident type 2 diabetes.

**Materials and Methods:**

Three repeated measures of BMI, WC and WHtR were collected over a decade (1991–2004) in 5666 participants without diabetes from the Whitehall II cohort study of British civil servants. Linear mixed‐effects models were used to estimate standardised individual change rates of the three indices, and participants were then followed up for incident diabetes until 2023. We examined the prospective associations between change rates of the three indices and diabetes by Cox regression analysis.

**Results:**

The mean change rates for BMI, WC and WHtR were 0.04, 0.06 and 0.07 standard deviation (SD)/year, respectively. There were 633 incident diabetes cases after a median follow‐up of 17.6 years. For every 1‐SD increase per decade, WC change (HR: 3.01, 95% CI: 2.15–4.22) was associated with a higher diabetes risk than changes in WHtR (HR: 2.67, 95% CI: 2.00–3.55), and BMI (HR: 1.98, 95% CI: 1.53–2.56). No significant interactions were found between the change rates and either sex or age.

**Conclusions:**

The changes in WC and WHtR over a decade were associated with a higher risk of developing diabetes than the change in BMI in this British adult population. Routine monitoring of WC in general practice may provide more benefit than BMI management alone in preventing diabetes.

## 1 INTRODUCTION

Type 2 diabetes is one of the fast‐growing epidemics and urgent health problems worldwide. According to the International Diabetes Federation's estimates, the global number of diagnosed cases of diabetes is expected to reach 643 million by 2030 and 783 million by 2045.^1^ In the UK, a survey showed that in 2018, around 4.7 million persons were diagnosed with diabetes, and 90% of those cases were type 2 diabetes.^2^

Obesity is widely recognised as a driving risk factor for the development of type 2 diabetes.^3^ It is an excess of body fat causing risk to health,^4^ most commonly evaluated by body mass index (BMI, expressed in body weight in kilograms divided by height in square metre). In the UK, BMI is routinely measured as part of the National Health Service Health Check, which is offered every 5 years to adults aged 40 years and above,^5^ and it is also measured in schools as part of the National Child Measurement Programme.^6^ However, a growing number of studies have demonstrated that using BMI solely is insufficient to assess obesity‐related risk, and other easily measurable indices, such as waist circumference (WC) and waist‐to‐height ratio (WHtR), may more accurately identify obesity‐related risks, especially for diabetes.^7^, ^8^, ^9^

Values of adiposity indices do not remain constant even during adulthood, especially in the face of the alarming increase in global obesity.^1^ However, many previous studies relied on a single measurement to represent an individual's weight (or WC) over time.^10^, ^11^ To address this limitation, we used repeated measures of three adiposity indices to more accurately reflect adiposity changes over time and their long‐term impact on health outcomes. Although some existing studies have examined changes in adiposity indices based on two time‐point measurements, particularly for BMI and WC,^12^, ^13^, ^14^ few have specifically focused on changes in WHtR, especially in light of the fact that several studies have found that elevated WHtR is more strongly associated with diabetes risk than BMI.^10^, ^15^ Moreover, as a waist‐based measure, WHtR is less influenced by sex and ethnicity than WC,^16^ which may contribute to its more consistent performance across different populations. Thus, by examining changes in WHtR alongside BMI and WC, our study helps fill this evidence gap. Additionally, previous studies involving multiple adiposity indices mostly examined changes in each index separately,^17^, ^18^ with few directly comparing the effects of their changes quantitatively. Our study addresses this gap by quantitatively comparing the effects of changes across these three indices. Evidence on adiposity changes in the British population is limited. Thus, our research, utilising an established British cohort, adds evidence for the middle‐aged British population. Also, most of the existing studies assessed changes in adiposity indices using absolute differences without consistently considering the duration of change. Our study explicitly used annual change rates (change per year), helping to standardise results and thus potentially enhancing comparability for future research.

Although there is evidence from observational studies that elevated adiposity indices are significantly associated with the development of diabetes,^12^, ^14^ the estimates for specific sex groups or age groups were seldom reported. A US study using the National Health and Nutrition Examination Survey has indicated that on the whole, adult women are more likely to be obese, but men have a higher risk of developing type 2 diabetes.^19^ The association between age and obesity and diabetes is complicated. Obesity is a common problem among older adults, but its prevalence decreases at advanced ages.^20^ On the other hand, the prevalence of diabetes increases progressively with age.^21^ Therefore, it remains unclear whether sex and age modify the association between changes in adiposity indicators and the risk of developing diabetes. Existing relevant studies rarely considered both sex and age, and current clinical recommendations also do not provide information on age‐specific or sex‐sensitive prevention strategies and management.^22^ Thus, we stratify participants into different subgroups with potentially differing diabetes risk. These findings may inform subgroup‐specific strategies in general practice by identifying groups with stronger associations between adiposity change and diabetes risk, where obesity monitoring and control may yield greater preventive effects.

Therefore, this study investigates change rates of BMI, WC and WHtR among British middle‐aged adults, and compares their associations with the development of diabetes. We also examine whether these associations are modified by age or sex.

## 2 MATERIALS AND METHODS

### 2.1 Data collection

We used data from the Whitehall II (WHII) study. The WHII is an ongoing cohort study of 10 308 British civil servants aged 35–55 years recruited from 20 London‐based offices starting in 1985–1988 (Phase 1), of whom 6895 (66.9%) were men.^23^ Phase 1 of WHII included a self‐administered questionnaire and a clinical examination to collect information covering demographics, lifestyle factors and health status. For the subsequent data collection, questionnaires alone were alternated with questionnaires accompanied by a clinical examination.^24^ The Ethics Committee of University College London approved the study. The use of human materials is in accordance with the Declaration of Helsinki.

### 2.2 Study population

**FIGURE 1:**
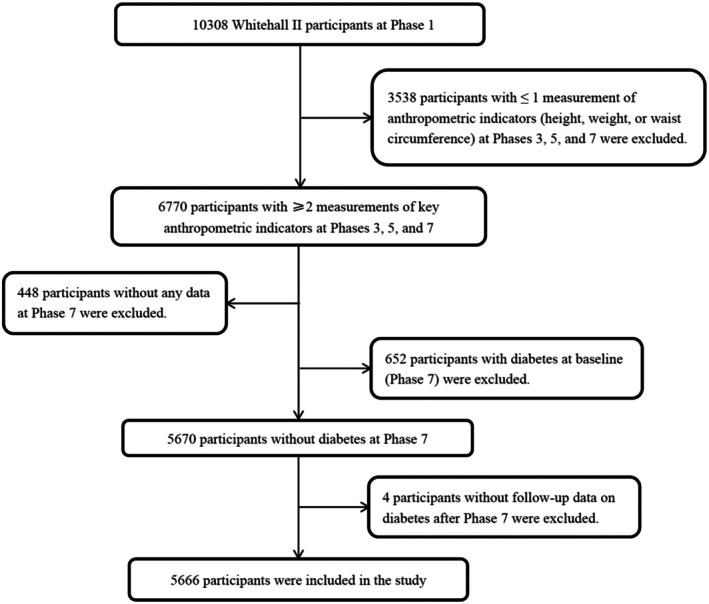
Flow chart of the included participants.

To obtain individual change rates of the three adiposity indicators (BMI, WC and WHtR) of the participants, we included subjects with repeated measurements of height, weight and WC. The anthropometric measurements were first collected at Phase 3 (1991–1994). The second and third anthropometric indicator surveys were in Phase 5 (1997–1999) and Phase 7 (2002–2004). Eligible participants had measurements (weight, height and WC) from at least two of the three phases (Phases 3, 5 and 7). To explore the prospective association between changes in adiposity indices and incident diabetes, we further excluded participants who already had diabetes at Phase 7 (the start of follow‐up for incident diabetes), as well as those without follow‐up data on diabetes after Phase 7. A flow chart of the included participants is demonstrated in Figure 1.

### 2.3 Adiposity indices

We studied three adiposity indices, including BMI (weight [kg]/height^2^ [m^2^]), WC (cm) and WHtR (WC [cm]/height [cm]). Weight was measured using a Soehnle electronic scale with a digital readout, with participants wearing only underwear. Height was measured using a stadiometer, with participants standing erect and the head in the Frankfurt plane. WC was defined as the smallest circumference at or below the costal margin, measured using a fibreglass tape measure with a tensile strength of 600 g.^25^

To enable quantitative comparison across different adiposity indices, we standardised BMI, WC and WHtR. The mean and standard deviation (SD) of each index were calculated at Phase 3. Repeated measures of BMI, WC and WHtR at Phases 3, 5 and 7 were then standardised individually using the following formula:

Standardised value = (raw value at Phase 3/5/7 − mean at Phase 3)/SD at Phase 3.

This method of standardising repeated measures has also been applied in previous studies using the Whitehall II dataset.^26^, ^27^

The exposure variables were the individual change rates of BMI, WC and WHtR, with calculation methods described in the statistical analysis section.

### 2.4 Covariates

For attenuating potential bias on initial weight or waist circumference (Phase 3) and estimating the effect of change of adiposity indicator on a health outcome (diabetes), included covariates were initial value of adiposity index (Phase 3), sex (female or male), ethnicity (white or non‐white), age at Phase 7 (continuous), education level (low, middle or high), family history of diabetes (yes or no) and some potential time‐varying confounders (from both Phase 3 and Phase 7), including smoking (never, occasional or current), drinking (non‐current, current moderate or current heavy), socioeconomic position (defined by either current or last recorded employment grade: low, intermediate or high),^28^ physical activity (inactive: <1 h/week of moderate and <1 h/week of vigorous activity; active: ≥2.5 h/week of moderate activity or ≥1 h/week of vigorous activity; moderate: those not meeting criteria for inactive or active),^29^ dietary behaviour (frequency of fruit and vegetables consumption per week: <daily or ≥daily),^28^ and menopause status (female subjects: yes or no). Directed acyclic graph (DAG) for the study variables is provided in Figure S1.

### 2.5 Outcome variable and follow‐up

The study outcome was type 2 diabetes, which was defined as a fasting blood glucose ≥7.0 mmol/L, a 2‐h postload glucose ≥11.1 mmol/L at clinical examination, physician‐diagnosed type 2 diabetes, use of diabetes medication, or hospital record of diabetes between 2002 (Phase 7) and March 2023.^30^, ^31^ For each individual, follow‐up time began on the participation date of Phase 7 and ended on the occurrence of diabetes, death, emigration or the end date of the last visit, whichever occurred first.

### 2.6 Statistical analysis

Continuous variables were presented as means ± SDs or median (25th–75th percentiles), and categorical variables as frequencies with proportions. Normality of continuous variables was assessed prior to group comparisons; two‐sided *t* tests were used for normally distributed variables, while Wilcoxon rank‐sum tests were applied for variables that were not normally distributed. Chi‐square tests were used to evaluate categorical variables.

We used linear mixed‐effects models to estimate the individual change rate per year for each adiposity indicator. We analysed both standardised values (used in the main analyses to enable quantitative comparison across indices with different units) and raw values (presented in the Supplementary material to facilitate interpretability). Individual change rate (per year) was estimated by allowing both a random slope and a random intercept for time. For each adiposity index separately, time was the independent variable (unit is year. The participation date of Phase 3 is defined as 0; the 2nd and 3rd time points are years from Phase 3 to Phase 5 and 7, respectively [if not missing]), and the repeatedly measured adiposity index (i.e., BMI/WC/WHtR) was the corresponding dependent variable. The formula is as follows:

Value of adiposity index=intercept+change rate×time.

Cox regression analysis was applied to estimate hazard ratios (HRs) and their 95% confidence interval (CI) for incident diabetes in relation to the change rate of different adiposity indices. The proportional hazards assumption was tested using Schoenfeld residuals. *p*‐Values from the global test (>0.05) indicated that the assumption was not violated. For each adiposity index separately, two Cox models were estimated. Model_1_ only included the change rate of the adiposity index and its initial value (Phase 3). For Model_2_, besides the variables of Model_1_, we additionally included several constant covariates, including sex, ethnicity, family history of diabetes, education level, and age (Phase 7). Also potential time‐varying covariates from both Phase 3 and Phase 7, including smoking status, drinking status, socioeconomic position, physical activity and dietary behaviour. We also conducted stratification analyses. In the sex stratification (male or female), the sex variable was excluded from Model_2_ for both male and female subgroups, but menopause status was additionally adjusted for in the female subgroup. In the age stratification (<60 or ≥60 years, median age of the sample at Phase 7), building on Model_2_, the age variable was excluded from both the <60 and ≥60 years subgroups.

Multiple imputation by chained equations was conducted to impute missing covariates (Table S1), with 30 imputed sets. To test the robustness of the findings, we also conducted sensitivity analyses examining the prospective associations between change rates of adiposity indices and incident diabetes among participants with complete data on the included covariates. To explore the influence of potential immortal time bias on the results, we estimated individual change rates (using raw values) of each adiposity index for the excluded subjects who were without diabetes at Phase 3 survey but had developed diabetes at Phase 7 survey, and then compared their individual change rates, as well as some basic characteristics, with those of the included subjects. All reported *p* values were two‐sided, and <0.05 indicates a statistical significance. All analyses in the study were performed by R (version 4.4.1).

## 3 RESULTS

### 3.1 Characteristics of participants

We included 5666 participants. Table 1 presents their baseline characteristics. The mean age at Phase 7 was 61.15 years (SD: 8.03), and 1643 (29.0%) were females. From Phase 3 to Phase 7, average BMI, WC and WHtR increased from 24.99 to 26.53 kg/m^2^, 83.07 to 90.72 cm and 0.48 to 0.53, respectively.

**TABLE 1:** Characteristics of the included participants.

Variable	Total, *N* (%), *N* = 5666 (100)
Age at Phase 7, mean ± SD, years	61.15 ± 8.03
≥60, *N* (%)	2850/5666 (50.3)
Sex (female), *N* (%)	1643/5666 (29.0)
Ethnicity (non‐white), *N* (%)	379/5660 (6.7)
BMI, kg/m^2^	
Phase 3, mean ± SD (ref)	24.99 ± 3.45
≥25, *N* (%)	2457/5490 (44.8)
Standardised value, median (interquartile range)	−0.11 (−0.65, 0.51)
Phase 5, mean ± SD	25.95 ± 3.84
Standardised value, median (interquartile range)	0.15 (−0.45, 0.82)
Phase 7, mean ± SD	26.53 ± 4.18
Standardised value, median (interquartile range)	0.29 (−0.35, 1.05)
WC, cm	
Phase 3, mean ± SD (ref)	83.07 ± 11.08
≥Cut‐off,^a^ *N* (%)	1704/5447 (31.3)
Standardised value, median (interquartile range)	0.03 (−0.67, 0.64)
Phase 5, mean ± SD	88.03 ± 11.48
Standardised value, median (interquartile range)	0.45 (−0.26, 1.10)
Phase 7, mean ± SD	90.72 ± 12.05
Standardised value, median (interquartile range)	0.70 (−0.04, 1.38)
WHtR	
Phase 3, mean ± SD (ref)	0.48 ± 0.06
≥0.5, *N* (%)	1951/5440 (35.9)
Standardised value, median (interquartile range)	−0.01 (−0.65, 0.63)
Phase 5, mean ± SD	0.51 ± 0.06
Standardised value, median (interquartile range)	0.45 (−0.20, 1.16)
Phase 7, mean ± SD	0.53 ± 0.07
Standardised value, median (interquartile range)	0.78 (0.07, 1.50)
Family history of diabetes, *N* (%)	549/5585 (9.8)
Education level	
Low	192/4190 (4.6)
Middle	2093/4190 (50.0)
High	1905/4190 (45.5)
Covariate at Phase 3	
Smoking status, *N* (%)	
Never smoker	2580/5240 (49.2)
Ex‐smoker	2045/5240 (39.0)
Current smoker	615/5240 (11.7)
Drinking status, *N* (%)	
Non‐current	907/5521 (16.4)
Current moderate	3221/5521 (58.3)
Current heavy	1393/5521 (25.2)
Fruit/vegetable consumption, *N* (%)	
<Daily	1986/5519 (36.0)
≥Daily	3533/5519 (64.0)
Physical activity	
Inactive	999/5525 (18.1)
Moderate	2011/5525 (36.4)
Active	2515/5525 (45.5)
Socioeconomic position, *N* (%)	
Low	669/5522 (12.1)
Intermediate	2342/5522 (42.4)
High	2511/5522 (45.5)
Covariate at Phase 7	
Smoking status, *N* (%)	
Never smoker	2763/5616 (49.2)
Ex‐smoker	2414/5616 (43.0)
Current smoker	439/5616 (7.8)
Drinking status, *N* (%)	
Non‐current	879/5553 (15.8)
Current moderate	2924/5553 (52.7)
Current heavy	1750/5553 (31.5)
Fruit/vegetable consumption, *N* (%)	
<Daily	1376/5574 (24.7)
≥Daily	4198/5574 (75.3)
Physical activity	
Inactive	380/5548 (6.8)
Moderate	629/5548 (11.3)
Active	4539/5548 (81.8)
Socioeconomic position, *N* (%)	
Low	630/5565 (11.3)
Intermediate	2513/5565 (45.2)
High	2422/5565 (43.5)

*Note*: Drinking status: current moderate (1–14 unit/week), current heavy (>14 unit/week); socioeconomic position: defined by either current or last recorded employment grade; physical activity: inactive (<1 h/week of moderate physical activity and <1 h/week of vigorous physical activity), active (>2.5 h/week of moderate physical activity or >1 h/week of vigorous physical activity), or moderately active (if not active or inactive); standardised BMI = (BMI at Phase * −24.99)/3.45; standardised WC = (WC at Phase * −83.07)/11.08; standardised WHtR = (WHtR at Phase * −0.48)/0.06.

Abbreviations: BMI, body mass index; SD, standard deviation; WC, waist circumference; WHtR, waist‐to‐height ratio.

^a^ Cut‐point of WC, ≥90 cm for men and ≥80 cm for women.

When the study population was stratified by sex, significant differences were observed in adiposity indices and several demographic and lifestyle characteristics. Additional details are provided in Table S2.

### 3.2 Change rates of adiposity indices

**FIGURE 2:**
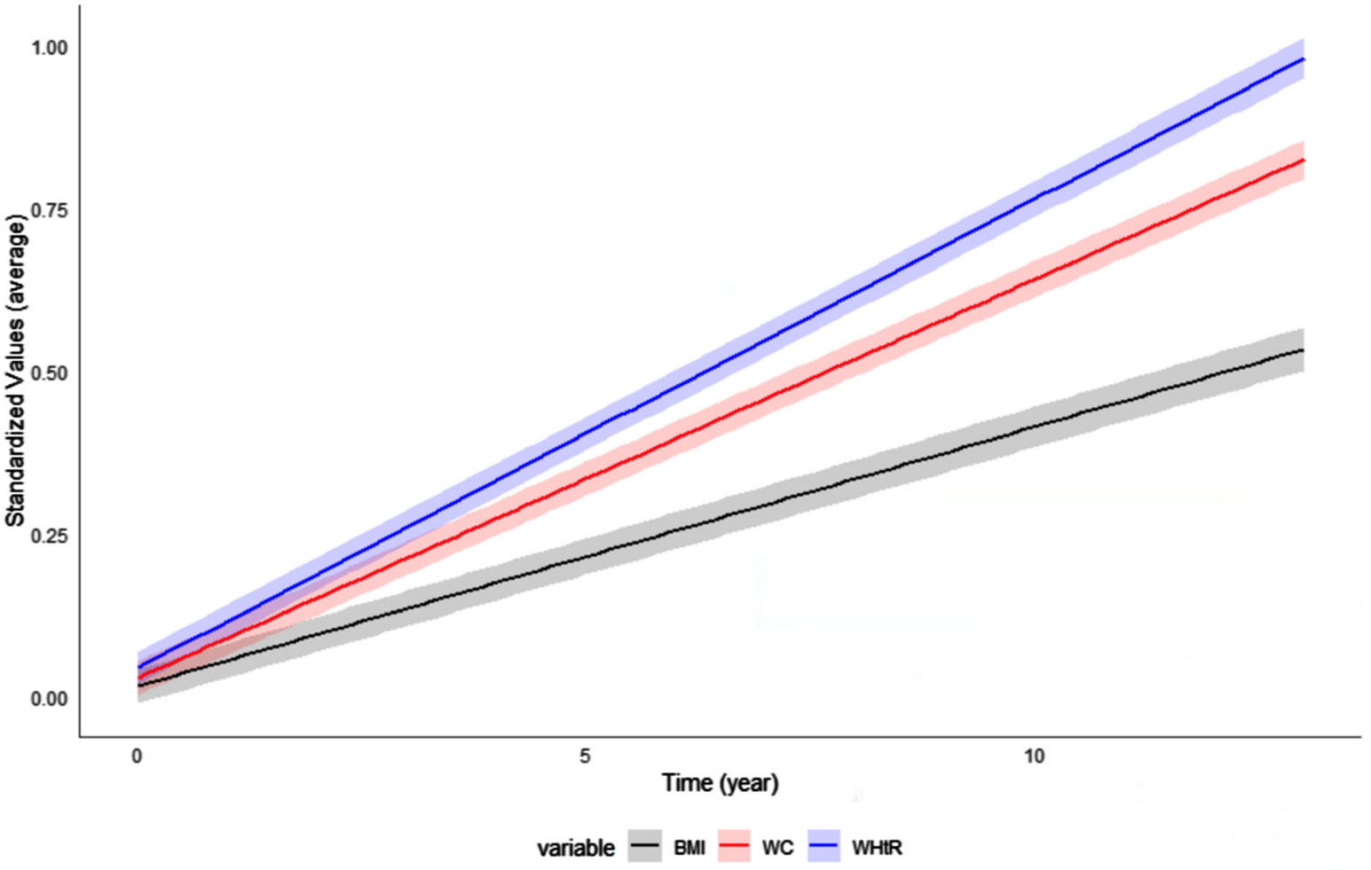
Unconditional estimated trajectories of average standardised adiposity indices from Phase 3 to Phase 7. Phase 3 is year 0; Average (±SD) follow‐up year at Phase 5 survey was 5.79 ± 0.53 years; Average (±SD) follow‐up year at Phase 7 survey was 11.20 ± 0.54 years. BMI, body mass index; CI, confidence interval; WC, waist circumference; WHtR, waist‐to‐height ratio.

Figure 2 illustrates the estimated trajectories of mean standardised BMI, WC and WHtR from Phase 3 to Phase 7. The average change rates for BMI, WC and WHtR were 0.04, 0.06 and 0.07 SD/year, respectively. Detailed results from mixed‐effects models are provided in Table S3. The correlation matrix between adiposity change rates and index values at Phases 3 and 7 are shown in Table S4.

Using linear mixed‐effects models, we estimated individual per‐year change rates (i.e., slopes) of the three adiposity indices, which were then used as exposure variables in subsequent analyses. Additional results based on the raw values of the indices are shown in Figure S2 and Table S5.

### 3.3 Association of change rates of adiposity indices with incident diabetes

Of the 5666 participants without diabetes at Phase 7, after a median follow‐up of 17.6 years (interquartile range: 16.8–18.2 years), 633 individuals developed diabetes. Table 2 (using standardised values) presents the prospective associations between the change rates (per 0.1‐SD increase per year) of adiposity indices and diabetes, as well as the effects of the initial (Phase 3) index values (per 1‐SD increase) on diabetes risk. For every 1‐SD increase over 10 years, WC change (adjusted HR [aHR]: 3.01, 95% CI: 2.15–4.22) was associated with a higher risk of developing diabetes than changes in WHtR (aHR: 2.67, 95% CI: 2.00–3.55) and BMI (aHR: 1.98, 95% CI: 1.53–2.56) in maximally adjusted models. Additionally, we found that initial WC (Phase 3) (aHR: 1.46, 95% CI: 1.34–1.59) and initial WHtR (aHR: 1.40, 95% CI: 1.30–1.52) were more strongly associated with diabetes risk compared with initial BMI (aHR: 1.27, 95% CI: 1.17–1.37). Results from complete cases (participants with no missing data for included covariates) also showed that the rate of WC change had the strongest association with diabetes risk among the three indicators (Table S6). Additional results based on the raw values of the indices are presented in Table S7.

**TABLE 2:** Hazard ratios for incident diabetes: Change rates of standardised adiposity indices and their initial values.

	Adjusted HR_1_ (95% CI)	Adjusted HR_2_ (95% CI)
Change rate^a^		
BMI	1.82 (1.43–2.32)	1.98 (1.53–2.56)
WC	2.98 (2.15–4.13)	3.01 (2.15–4.22)
WHtR	2.59 (1.97–3.40)	2.67 (2.00–3.55)
Initial value (Phase 3)^b^		
BMI	1.30 (1.21–1.40)	1.27 (1.17–1.37)
WC	1.49 (1.38–1.62)	1.46 (1.34–1.59)
WHtR	1.52 (1.42–1.64)	1.40 (1.30–1.52)

*Note*: Sample size = 5666, number of events = 633, imputation for the missing covariates; adjusted hazard ratio_1_ (adjusted HR_1_), the Cox regression model included change rate of standardised adiposity index (BMI or WC or WHtR), and corresponding standardised initial value of the adiposity indicator; adjusted hazard ratio_2_ (adjusted HR_2_), the Cox regression model included change rate of standardised adiposity index (BMI or WC or WHtR), corresponding standardised initial value of the adiposity indicator, sex, ethnicity, age at Phase 7, family history of diabetes, education level and covariates from both Phase 3 and Phase 7, including smoking, drinking, socioeconomic position, physical activity and dietary behaviour.

Abbreviations: BMI, body mass index; WC, waist circumference; WHtR, waist‐to‐height ratio.

^a^ Hazard ratio (per 0.1‐SD increase per year) for incident diabetes.

^b^ Hazard ratio (per 1‐SD increase) for incident diabetes.

### 3.4 Stratification analyses

**FIGURE 3:**
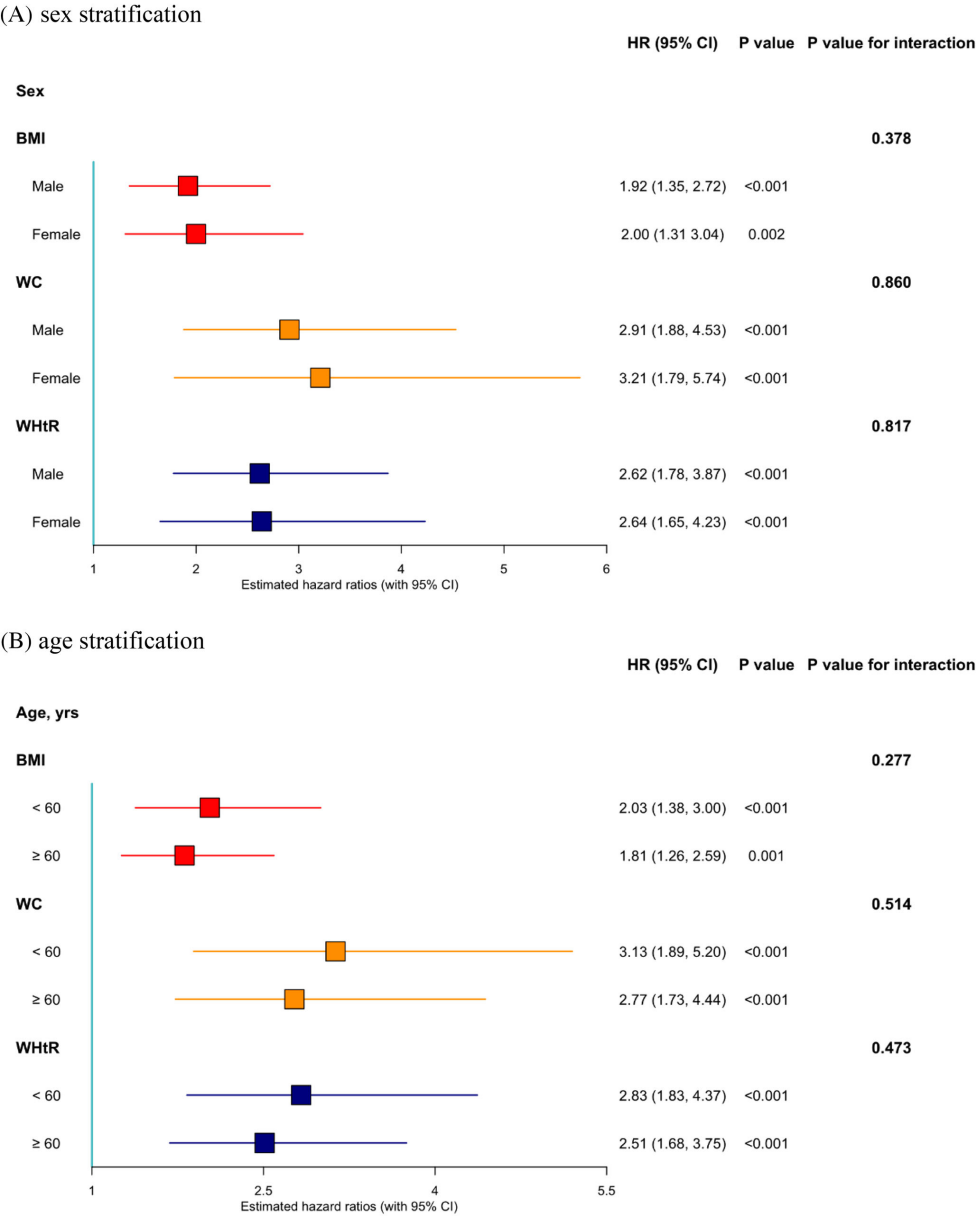
Subgroup analyses for incident diabetes in relation to change rates of different standardised adiposity indices. BMI, body mass index; CI, confidence interval; HR, hazard ratio; WC, waist circumference; WHtR, waist‐to‐height ratio.

As shown in Figure 3 and Table S8, there were no statistically significant interactions between the change rate of the three indicators and either sex or age in relation to incident diabetes (*p* ≥ 0.05). For each index, in the sex stratification, the HRs for the male and female subgroups were very similar. In the age stratification, the HRs for the <60 years subgroup were slightly higher than those for the ≥60 years subgroup. Additional results based on the raw values of the indices are shown in Table S9.

### 3.5 Analyses for the excluded persons (potential immortal time bias)

As shown in Table S10, there were 476 (34.7% females) excluded subjects who did not have diabetes at Phase 3 but had developed diabetes at the Phase 7 survey. Significant differences were observed in age, sex and the initial values (Phase 3) of adiposity indices between the excluded individuals and our study population; however, there were no statistically significant differences in the average change rates of the three indices (i.e., exposures) between the two groups.

## 4 DISCUSSION

The present study investigated the change rates of BMI, WC and WHtR separately in British civil servants and compared the effects of the change rates in different adiposity indices on incident diabetes. The results showed that WHtR and WC had higher average change rates than BMI in our study population. For every 1‐SD increase over 10 years, changes in WC and WHtR were associated with a higher risk of developing diabetes than changes in BMI. These findings suggest that routine monitoring of WC, in addition to BMI, may be helpful for identifying individuals at increased risk of type 2 diabetes in British middle‐aged adults.

First, our finding of a continuous increase in the average value of adiposity indices from 1991 to 2004 is consistent with trends reported in most previous studies and forms the foundation of this study. For instance, a study using data from the China Health and Nutrition Survey reveals significant increases in BMI during 1991–2011 for the cohort born in 1960–1969.^32^ In terms of WC, studies from the United States (with follow‐up from 1988 to 2000) and Australia (1992–2007) both reported continuous increases in WC over their respective follow‐up periods.^33^, ^34^ Additionally, the present study demonstrated an overall upward trend in WHtR in our subjects during the follow‐up. Although long‐term changes in WHtR have been less frequently studied than those in BMI and WC, some studies have reported increasing WHtR trends similar to those observed in our study.^35^, ^36^ Compared with existing studies, a key innovation of our study is that we directly compared change rates across multiple adiposity indices (BMI, WC and WHtR), rather than analysing them separately only. We found that, on average, WC and WHtR changed more rapidly than BMI among our participants during 1991–2004. This comparative analysis has been rarely reported in previous studies. The results may reflect a shift toward more prominent abdominal obesity in recent decades, indicating that abdominal obesity should receive greater attention in clinical and public health settings.

Second, our findings indicate that changes in WC and WHtR were more strongly associated with incident diabetes than the change in BMI, although all three indices showed statistically significant associations. Some previous studies have separately investigated the associations between changes in BMI or WC and diabetes, while comparisons across indices were not performed in those studies. For example, a study using data from the U.S. Health and Retirement Study cohort suggested that rapid increases in BMI were associated with a higher risk of incident diabetes compared with slower increases.^37^ Similarly, a UK cohort study showed that rising BMI trends were associated with increased diabetes risk.^38^ These findings from both studies are consistent with the association observed in our study. As for WC change, a population‐based cohort study from Japan showed that participants in the highest tertile of WC change had a significantly higher risk of developing diabetes than those in the lower tertiles.^39^ A similar association was observed in another cohort study, where greater WC increases were also linked to higher diabetes risk,^40^ consistent with our findings. However, a Danish study reported different findings: changes in WC absolute value were not significantly associated with diabetes risk among men, suggesting that WC change measured at only two time points may be insufficient to predict incident diabetes.^13^ This supports the rationale for our use of change rate, which accounts for both the amount of change and the time over which it occurred. The non‐significant result in the Danish study may partly be explained by the relatively small absolute changes in WC among male participants. This interpretation is further supported by another cohort study, which found a significant association with diabetes only in the group with the largest WC gain (≥14.6 cm).^41^

While several studies have separately examined changes in BMI or WC in relation to incident diabetes, studies comparing the strengths of these associations across different indices remain relatively limited. Among those that made such comparisons, some reported stronger associations for WC change than for BMI change,^40^, ^42^ which is consistent with our findings. Hence, we inferred that WC change may more sensitively reflect the diabetes risk associated with increased adiposity than BMI change. This may be explained by WC being more closely related to visceral fat than BMI, which is strongly associated with diabetes risk.^43^ Notably, studies examining such comparisons have rarely been conducted in British populations. Therefore, differences in populations should be considered when comparing findings from previous studies with our results. Nonetheless, this highlights that our study helps fill this gap by providing evidence from a well‐established British cohort. In addition, our study showed that the HR for the change rate in WC was relatively higher than that of WHtR. Although the reason remains unclear, one possible explanation is that the change rate captures the pace of fat accumulation, whereas WC and WHtR values represent abdominal obesity at a single point in time. In this context, the height‐adjustment advantage of WHtR in cross‐sectional analyses becomes less relevant, as the fat accumulation rate in adults is typically independent of height. WC change may therefore more directly reflect longitudinal increases in abdominal fat, resulting in a stronger association with diabetes risk. Although the HR for the change rate of WHtR was lower than that of WC in the present study, it was still higher than that for BMI. Our study also helps address an important research gap by providing evidence on the association between changes in WHtR and incident diabetes in a British population. Therefore, given the stronger association of waist‐based measures with incident diabetes, our findings suggest that routine tracking of WC changes might enhance diabetes risk assessment beyond relying solely on BMI in clinical and public health settings. In contrast with previous studies, another key innovation of our study is the direct quantitative comparison of the associations between changes in three adiposity indices and incident diabetes. By using the rate of change, which incorporates both the magnitude and duration of change, our approach also facilitates cross‐study comparisons, including those with varying follow‐up durations, and may provide a foundation for future research.

Third, in our stratification analyses, we found little evidence of interactions between the change rates of adiposity indices and either sex or age. This suggests that in our study populations, effects of the growth rate of the indices on diabetes development may be similar in males and females, and in subjects below and above 60 years of age. For this result, we speculate that it may be related to the following: (1) Regarding sex stratification, by Phase 7, the majority of our female participants (91.7%) were postmenopausal, with significantly reduced oestrogen levels. As oestrogen largely drives sex differences in health outcomes, this may explain why sex did not act as a modifier in our study. (2) For age stratification, although the mean age of the <60 and >60 groups differed by about 10 years (55.8 vs. 65.9 years), all participants were over 50 at Phase 7, falling within a middle‐to‐older age range. This appears to attenuate age‐related differences, which might explain why no significant interaction between adiposity change rates and age was found. Thus, further research is essential, particularly in wider age ranges that include younger individuals.

Although our study did not involve formal risk prediction modelling, our findings highlight the clinical value of dynamically measuring adiposity indices (e.g., repeated WC) for identifying populations at greater risk of diabetes. Some studies suggest that incorporating repeated anthropometric measures into dynamic prediction models may improve diabetes risk prediction compared with traditional static models.^44^, ^45^ For instance, a study showed that integrating longitudinal WC measurements may improve the calibration of diabetes risk prediction compared with baseline‐only measures.^45^ While such modelling was beyond our study's scope, our findings support the clinical utility of routinely tracking adiposity changes over time. Future studies are needed to explore dynamic adiposity measures in diabetes risk prediction.

Finally, beyond the earlier‐discussed innovations, a further strength of our study lies in its long‐term design: adiposity indices were repeatedly measured over a 10‐year period, and participants were then followed for incident diabetes for more than 15 years. The extended follow‐up duration may help enhance the stability and reliability of the observed associations. This approach allows for the assessment of the effects of adiposity change over time, in contrast with previous cohort studies that relied on a single‐point measurement. By reflecting dynamic changes in adiposity, our study more accurately reflects real‐world obesity trends and their long‐term impact on diabetes risk.

This study has some limitations. First, from Phase 7 of Whitehall II, the study populations participating in the follow‐up (survey for incident diabetes status) were over 50 years; therefore, our results may not apply to the younger adults. Second, the period between the first and third measurements of the adiposity index spanned over 10 years, and individuals with diabetes diagnosed prior to Phase 7 were excluded. Significant differences in certain baseline characteristics between excluded and included subjects may result in potential immortal time bias. Third, adiposity change rates were estimated using two or three repeated measurements, which is the minimum required to fit a mixed‐effects model with random intercepts and slopes. However, the number of measurements may still be considered limited, as approximately 30% of participants had only two data points. This may introduce variability in individual estimates. This limitation results from the data collection schedule of the Whitehall II study, which included only three relevant clinical assessments during the study period. Nevertheless, our use of mixed‐effects models remains a robust analytical approach for repeated measurements, widely adopted even with relatively limited numbers of observations. Future work using more frequent measurements may improve precision. Fourth, over 90% of the participants in the Whitehall II study were White. Therefore, due to the small sample size of non‐White people, we were unable to provide very precise analytical results on different ethnicities. Finally, our data, solely from British civil servants, may limit the generalisability of findings to other populations. Further study involving other populations is warranted.

## 5 CONCLUSIONS

The mean change rates of WHtR and WC over a decade were larger than that of BMI in this British adult population. For every 1‐SD increase per decade, changes in WC and WHtR were associated with a higher risk of developing diabetes than the change in BMI. Routine monitoring of WC in general practice may be an effective strategy for preventing type 2 diabetes.

## AUTHOR CONTRIBUTIONS

C.H. conducted conceptualisation, formal analysis, methodology, software, validation, visualisation and writing—original draft and editing. Y.C. carried out conceptualisation, methodology, supervision, validation and writing—review and editing. A.B. conducted methodology, project administration, resources, supervision, validation and writing—review and editing.

## FUNDING INFORMATION

The Whitehall II study is supported by the National Institute on Aging, NIH (R01AG056477, R01AG062553); the UK Medical Research Council (R024227, S011676); and the Wellcome Trust (221854/Z/20/Z). However, the authors did not receive direct financial support from these sources.

## CONFLICT OF INTEREST STATEMENT

The authors declare no conflicts of interest.

## PEER REVIEW

The peer review history for this article is available at https://www.webofscience.com/api/gateway/wos/peer‐review/10.1111/dom.16615.

## Supporting information


**Data S1.** Supporting information.

## Data Availability

The Whitehall II study data supporting the results of this manuscript are available at: https://www.ucl.ac.uk/psychiatry/research/mental-health-older-people/whitehall-ii/data-sharing.
