# Supplementary material for: Changes in adiposity indices over 10 years and risk of type 2 diabetes: The Whitehall II cohort study
Source: Diabetes Obes Metab. 2025 Jul 14;27(10):5664–74. doi: 10.1111/dom.16615 (PMC12409207; doi:10.1111/dom.16615)
Supplement: Supplementary file 1 — Data S1. Supporting information. [file DOM-27-5664-s001.docx]

**Table S1 Missingness in the covariates**

| **Variable** | **Participants with complete**  **exposures and outcome (N = 5666)**  **Missing, N (%)** |
| --- | --- |
| **Age at Phase 7** | 0 (0) |
| **Sex** | 0 (0) |
| **Ethnicity** | 6 (0.1) |
| **Education level** | 1476 (26.1) |
| **Family history of diabetes** | 81 (1.4) |
| **Initial BMI (Phase 3)** | 176 (3.1) |
| **Initial WC (Phase 3)** | 219 (3.9) |
| **Initial WHtR (Phase 3)** | 226 (4.0) |
| **Covariates at Phase 3** |  |
| **Smoking** | 426 (7.5) |
| **Drinking** | 145 (2.6) |
| **Socioeconomic position** | 144 (2.5) |
| **Physical activity** | 141 (2.5) |
| **Dietary behavior** | 147 (2.6) |
| **Menopause status (for female)** | 46/1643 (2.8) |
| **Covariates at Phase 7** |  |
| **Smoking** | 50 (0.9) |
| **Drinking** | 113 (2.0) |
| **Socioeconomic position** | 101 (1.8) |
| **Physical activity** | 118 (2.1) |
| **Dietary behavior** | 92 (1.6) |
| **Menopause status (for females)** | 69/1643 (4.2) |

BMI, body mass index; WC, waist circumference; WHtR, waist-to-height ratio

**Table S2 Characteristics of the included participants by sex**

| **Variable** | **Male, N (%)**  **N = 4023 (71.0)** | **Female, N (%)**  **N = 1643 (29.0)** | **P value**^†^ |
| --- | --- | --- | --- |
| **Age at Phase 7,** mean±SD, **yrs** | 60.79 ± 5.88 | 61.10± 5.97 | 0.074 |
| ≥ 60, N (%) | 1982/4023 (49.3) | 868/1643 (52.8) | 0.016 |
| **Ethnicity (White), N (%)** | 3828/4020 (95.2) | 1453/1640 (88.6) | <0.001 |
| **BMI, kg/m^2^** |  |  |  |
| Phase 3, mean±SD (ref) | 24.91 ± 2.98 | 25.18 ± 4.40 | 0.025 |
| ≥25, N (%) | 1761/3908 (45.1) | 696/1582 (44.0) | 0.490 |
| Standardized value  median (interquartile range) | -0.09 (-0.59, 0.48) | -0.17 (-0.83, 0.67) | 0.025 |
| Phase 5, mean±SD | 25.87 ± 3.36 | 26.14 ± 4.81 | 0.063 |
| Standardized value  median (interquartile range) | 0.17 (-0.38, 0.77) | 0.08 (-0.59, 1.00) | 0.063 |
| Phase 7, mean±SD | 26.41 ± 3.67 | 26.81± 5.22 | 0.006 |
| Standardized value  median (interquartile range) | 0.32 (-0.29, 0.96) | 0.26 (-0.49, 1.34) | 0.006 |
| **WC, cm** |  |  |  |
| Phase 3, mean±SD (ref) | 86.71±8.87 | 74.12±10.86 | <0.001 |
| ≥cut-off,^‡^ N (%) | 1307/3872 (33.8) | 397/1575 (25.2) | <0.001 |
| Standardized value  median (interquartile range) | 0.27 (-0.22, 0.82) | -0.95 (-1.50, -0.28) | <0.001 |
| Phase 5, mean±SD | 91.38±9.73 | 80.12±11.43 | <0.001 |
| Standardized value  median (interquartile range) | 0.70 (0.15, 1.27) | -0.43 (-1.02, 0.36) | <0.001 |
| Phase 7, mean±SD | 93.82±10.40 | 83.00±12.40 | <0.001 |
| Standardized value  median (interquartile range) | 0.91 (0.33, 1.54) | -0.17 (-0.82, 0.70) | <0.001 |
| **WHtR** |  |  |  |
| Phase 3, mean±SD (ref) | 0.49±0.05 | 0.46±0.07 | <0.001 |
| ≥0.5, N (%) | 1581/3866 (40.9) | 370/1574 (23.5) | <0.001 |
| Standardized value  median (interquartile range) | 0.14 (-0.40, 0.71) | -0.56 (-1.17, 0.27) | <0.001 |
| Phase 5, mean±SD | 0.52±0.06 | 0.49±0.07 | <0.001 |
| Standardized value  median (interquartile range) | 0.57 (0.01, 1.22) | 0.02 (-0.69, 0.99) | <0.001 |
| Phase 7, mean±SD | 0.54±0.06 | 0.51±0.08 | <0.001 |
| Standardized value  median (interquartile range) | 0.87 (0.25, 1.54) | 0.45 (-0.40, 1.37) | <0.001 |
| **Family history of Diabetes, N (%)** | 362/3969 (9.1) | 187/1616 (11.6) | 0.006 |
| **Education level** |  |  | 0.003 |
| Low | 133/3004 (4.4) | 59/1186 (5.0) |  |
| Middle | 1456/3004 (48.5) | 637/1186 (53.7) |  |
| High | 1415/3004 (47.1) | 490/1186 (41.3) |  |
| **Covariate at Phase 3** |  |  |  |
| **Smoking status, N (%)** |  |  | <0.001 |
| Never smoker | 1767/3740 (47.2) | 813/1500 (54.2) |  |
| Ex-smoker | 1579/3740 (42.2) | 466/1500 (31.1) |  |
| Current smoker | 394/3740 (10.5) | 221/1500 (14.7) |  |
| **Drinking status, N (%)** |  |  | <0.001 |
| Non-current | 490/3921 (12.5) | 417/1600 (26.1) |  |
| Current moderate | 2202/3921 (56.2) | 1019/1600 (63.7) |  |
| Current heavy | 1229/3921 (31.3) | 164/1600 (10.2) |  |
| **Fruit/vegetable consumption, N (%)** |  |  | <0.001 |
| < Daily | 1496/3919 (38.2) | 490/1600 (30.6) |  |
| ≥ Daily | 2423/3919 (61.8) | 1110/1600 (69.4) |  |
| **Physical activity** |  |  | <0.001 |
| Inactive | 481/3924 (12.3) | 518/1601 (32.4) |  |
| Moderate | 1452/3924 (37.0) | 559/1601 (34.9) |  |
| Active | 1991/3924 (50.7) | 524/1601 (32.7) |  |
| **Socioeconomic position, N (%)** |  |  | <0.001 |
| Low | 171/3922 (4.4) | 498/1600 (31.1) |  |
| Intermediate | 2023/3922 (51.6) | 319/1600 (19.9) |  |
| High | 1728/3922 (44.1) | 783/1600 (48.9) |  |
| **Menopause (Yes, only female)** | NA | 749/1597 (46.9) | NA |
| **Covariate at Phase 7** |  |  |  |
| **Smoking status, N (%)** |  |  | <0.001 |
| Never smoker | 1865/3989 (46.8) | 898/1627 (55.2) |  |
| Ex-smoker | 1838/3989 (46.1) | 576/1627 (35.4) |  |
| Current smoker | 286/3989 (7.2) | 153/1627 (9.4) |  |
| **Drinking status, N (%)** |  |  | <0.001 |
| Non-current | 440/3955 (11.1) | 439/1598 (27.5) |  |
| Current moderate | 1988/3955 (50.3) | 936/1598 (58.6) |  |
| Current heavy | 1527/3955 (38.6) | 223/1598 (14.0) |  |
| **Fruit/vegetable consumption, N (%)** |  |  | <0.001 |
| < Daily | 1034/3967 (26.1) | 342/1607 (21.3) |  |
| ≥ Daily | 2933/3967 (73.9) | 1265/1607 (78.7) |  |
| **Physical activity** |  |  | <0.001 |
| Inactive | 212/3950 (5.4) | 168/1598 (10.5) |  |
| Moderate | 377/3950 (9.5) | 252/1598 (15.8) |  |
| Active | 3361/3950 (85.1) | 1178/1598 (73.7) |  |
| **Socioeconomic position, N (%)** |  |  | <0.001 |
| Low | 163/3950 (4.1) | 467/1615 (28.9) |  |
| Intermediate | 2149/3950 (54.4) | 364/1615 (22.5) |  |
| High | 1638/3950 (41.5) | 784/1615 (48.5) |  |
| **Menopause (Yes, only female)** | NA | 1443/1574 (91.7) | NA |

^†^ T-test or Wilcoxon rank-sum test for continuous variables and chi-square test for categorical variables;

^‡^ Cut-point of WC, **≥**90 cm for men and **≥**80 cm for women;

BMI, body mass index; WC, waist circumference; WHtR, waist-to-height ratio; Drinking: current moderate (1-14 unit/w), current heavy (>14 unit/w); Socioeconomic position: defined by either current or last recorded employment grade; Physical activity: inactive (<1 hr/week of moderate physical activity and <1 hr/week of vigorous physical activity), active (>2.5 hrs/week of moderate physical activity or >1 hr/week of vigorous physical activity), or moderately active (if not active or inactive);

Standardized BMI = (BMI at Phase * - 24.99) / 3.45;

Standardized WC = (WC at Phase * - 83.07) / 11.08;

Standardized WHtR = (WHtR at Phase * - 0.48) / 0.06.

**Table S3 Results of mixed effects models (using standardized values of BMI, WC, and WHtR)**

| **Adiposity index** |  |  |  |
| --- | --- | --- | --- |
| BMI |  |  |  |
| *Fixed* | *Estimate* | *SE* | *95% CI* |
| Intercept | 0.017 | 0.013 | (-0.009, 0.043) |
| Time | *0.040* ^***^ | 0.001 | (0.038, 0.041) |
| *Random* | *Variance* | *SD* | *Correlation* |
| Intercept | 0.942 | 0.971 |  |
| Time | 0.002 | 0.040 | 0.33 |
| *Average change rate, SD/year, IQR* | 0.04 (0.02, 0.06) |  |  |
| Male | 0.04 (0.02, 0.05) |  |  |
| Female | 0.04 (0.02, 0.06) |  |  |
| **WC** |  |  |  |
| *Fixed* | *Estimate* | *SE* | *95% CI* |
| Intercept | 0.030 ^*^ | 0.013 | (0.004, 0.056) |
| Time | *0.061* ^***^ | 0.001 | (0.060, 0.063) |
| *Random* | *Variance* | *SD* | *Correlation* |
| Intercept | 0.906 | 0.952 |  |
| Time | 0.001 | 0.035 | 0.05 |
| *Average change rate, SD/year, IQR* | 0.06 (0.05, 0.07) |  |  |
| Male | 0.06 (0.05, 0.07) |  |  |
| Female | 0.06 (0.04, 0.08) |  |  |
| **WHtR** |  |  |  |
| *Fixed* | *Estimate* | *SE* | *95% CI* |
| Intercept | 0.045 ^***^ | 0.013 | (0.020, 0.071) |
| Time | *0.072* ^***^ | 0.001 | (0.070, 0.074) |
| *Random* | *Variance* | *SD* | *Correlation* |
| Intercept | 0.850 | 0.922 |  |
| Time | 0.002 | 0.039 | 0.13 |
| *Average change rate, SD/year, IQR* | 0.07 (0.05, 0.09) |  |  |
| Male | 0.07 (0.05, 0.08) |  |  |
| Female | 0.07 (0.05, 0.10) |  |  |

BMI, body mass index; WC, waist circumference; WHtR, waist-to-height ratio; SE, standard error; SD, standard deviation; CI, confidence interval; IQR, interquartile range

^***^, P<0.001; ^**^, P<0.01; ^*^, P<0.05

**Table S4 Correlation matrix for correlations between the rate of change in adiposity indicators and their values at Phases 3 and 7**

1. BMI

|  | **BMI at Phase 3** | **Change rate of BMI** | **BMI at Phase 7** |
| --- | --- | --- | --- |
| **BMI at Phase 3** | - |  |  |
| **Change rate of BMI** | 0.40 | - |  |
| **BMI at Phase 7** | 0.87 | 0.80 | - |

1. WC

|  | **WC at Phase 3** | **Change rate of WC** | **WC at Phase 7** |
| --- | --- | --- | --- |
| **WC at Phase 3** | - |  |  |
| **Change rate of WC** | 0.07 | - |  |
| **WC at Phase 7** | 0.84 | 0.59 | - |

1. WHtR

|  | **WHtR at Phase 3** | **Change rate of WHtR** | **WHtR at Phase 7** |
| --- | --- | --- | --- |
| **WHtR at Phase 3** | - |  |  |
| **Change rate of WHtR** | 0.17 | - |  |
| **WHtR at Phase 7** | 0.82 | 0.71 | - |

BMI, body mass index; WC, waist circumference; WHtR, waist-to-height ratio

**Table S5 Results of mixed effects models (using raw values of BMI, WC, and WHtR)**

| **Adiposity index** |  |  |  |
| --- | --- | --- | --- |
| **BMI, kg/m^2^** |  |  |  |
| *Fixed* | *Estimate* | *SE* | *95% CI* |
| Intercept | 25.050^***^ | 0.046 | (24.960, 25.140) |
| Time | *0.137* ^***^ | 0.002 | (0.132, 0.142) |
| *Random* | *Variance* | *SD* | *Correlation* |
| Intercept | 11.217 | 3.349 |  |
| Time | 0.019 | 0.137 | 0.33 |
| *Average change rate, kg/m^2^/year, IQR* | 0.12 (0.07, 0.19) |  |  |
| Male | 0.12 (0.07, 0.18) |  |  |
| Female | 0.13 (0.06, 0.21) |  |  |
| **WC, cm** |  |  |  |
| *Fixed* | *Estimate* | *SE* | *95% CI* |
| Intercept | 83.400 ^***^ | 0.147 | (83.112, 83.688) |
| Time | *0.678 ^***^* | 0.008 | (0.663, 0.693) |
| *Random* | *Variance* | *SD* | *Correlation* |
| Intercept | 111.237 | 10.547 |  |
| Time | 0.147 | 0.383 | 0.05 |
| *Average change rate, cm/year, IQR* | 0.66 (0.51, 0.83) |  |  |
| Male | 0.66 (0.52, 0.82) |  |  |
| Female | 0.65 (0.49, 0.85) |  |  |
| **WHtR** |  |  |  |
| *Fixed* | *Estimate* | *SE* | *95% CI* |
| Intercept | 0.4827 *^***^* | 0.0008 | (0.4812, 0.4842) |
| Time | *0.0043 ^***^* | 0.00005 | (0.0042, 0.0044) |
| *Random* | *Variance* | *SD* | *Correlation* |
| Intercept | 0.0031 | 0.0553 |  |
| Time | <0.0001 | 0.0023 | 0.13 |
| *Average change rate, per year, IQR* | 0.004 (0.003, 0.005) |  |  |
| Male | 0.004 (0.003, 0.005) |  |  |
| Female | 0.004 (0.003, 0.006) |  |  |

BMI, body mass index; WC, waist circumference; WHtR, waist-to-height ratio; SE, standard error; SD, standard deviation; CI, confidence interval.

^***^, P<0.001; ^**^, P<0.01; ^*^, P<0.05

**Table S6 Estimated hazard ratios for incident diabetes in relation to the change rates of standardized adiposity indicators and their initial values (Phase 3) in complete data**

|  | **Adjusted HR_2_**  **(95% CI)** | **P value** |
| --- | --- | --- |
| **Change rate**^†^ |  |  |
| BMI | 1.68 (1.21-2.33) | 0.002 |
| WC | 2.62 (1.72-4.01) | <0.001 |
| WHtR | 2.31 (1.60-3.33) | <0.001 |
| **Initial value (Phase 3)**^‡^ |  |  |
| BMI | 1.33 (1.20-1.46) | <0.001 |
| WC | 1.49 (1.33-1.66) | <0.001 |
| WHtR | 1.42 (1.29-1.57) | <0.001 |

BMI, body mass index; WC, waist circumference; WHtR, waist-to-height ratio;

^†^ Hazard ratio (Per 0.1‐SD Increase per year) for incident diabetes;

^‡^ Hazard ratio (Per 1‐SD Increase) for incident diabetes;

Sample size = 3681, number of events = 395;

Adjusted hazard ratio_2_, the Cox regression model included change rate of standardized adiposity index (BMI or WC or WHtR), corresponding standardized initial value of the adiposity indicator, sex, ethnicity, age at Phase 7, family history of diabetes, education level, and covariates from both Phase 3 and Phase 7, including smoking, drinking, socioeconomic position, physical activity, and dietary behavior.

**Table S7 Estimated hazard ratios for incident diabetes in relation to the change rates of adiposity indicators and their initial (Phase 3) values (using raw values of the indicators)**

|  | **Adjusted HR_1_**  **(95% CI)** | **Adjusted HR_2_**  **(95% CI)** |
| --- | --- | --- |
| **Change rate** |  |  |
| BMI | 5.71 (2.84-11.48) | 7.26 (3.45-15.26) |
| WC | 2.68 (1.99-3.60) | 2.71 (2.00-3.67) |
| WHtR^†^ | 4.59 (2.89-7.27) | 4.87 (3.00-7.89) |
| **Initial values (Phase 3)** |  |  |
| BMI | 1.08 (1.06-1.10) | 1.07 (1.05-1.10) |
| WC | 1.04 (1.03-1.04) | 1.03 (1.03-1.04) |
| WHtR^‡^ | 1.08 (1.06-1.09) | 1.06 (1.05-1.07) |

^†^ Hazard ratio (Per 0.01 Increase per year) for incident diabetes;

^‡^ Hazard ratio (Per 0.01 Increase) for incident diabetes;

HR, hazard ratio; CI, confidence interval; BMI, body mass index; WC, waist circumference; WHtR, waist-to-height ratio;

Sample size = 5666, number of events = 633, imputation for the missing covariates;

Adjusted hazard ratio_1_, the Cox regression model included change rate of adiposity index (BMI or WC or WHtR), and corresponding initial value of the adiposity indicator;

Adjusted hazard ratio_2_, the Cox regression model included change rate of adiposity index (BMI or WC or WHtR), corresponding initial value of the adiposity indicator, sex, ethnicity, age at Phase 7, family history of diabetes, education level, and covariates from both Phase 3 and Phase 7, including smoking, drinking, socioeconomic position, physical activity, and dietary behavior.

**Table S8 Cumulative event (diabetes) rates for different subgroups at the end of follow-up**

| **Subgroup** | **N (%)** | **Diabetes, N (%)** | **Median follow-up time, yrs** | **P value** |
| --- | --- | --- | --- | --- |
| **Sex** |  |  |  | 0.015 |
| Male | 4023 (71.0) | 476 (11.8) | 17.4 |  |
| Female | 1643 (29.0) | 157 (9.6) | 17.8 |  |
| **Age, yrs** |  |  |  | <0.001 |
| < 60 | 2816 (49.7) | 267 (9.5) | 17.6 |  |
| ≥60 | 2850 (50.3) | 366 (12.8) | 17.6 |  |

P value for the differences in number of event (diabetes) between the different subgroups

**Table S9 Subgroup analyses for diabetes in relation to the change rates of different adiposity indicators (using raw values of adiposity indicators)**

| **Stratification** | **HR (95% CI)** | **P value** | **P value for interaction** |
| --- | --- | --- | --- |
| **Sex** | | | |
| **Change rate of BMI** |  |  | 0.377 |
| Male | 6.61 (2.40-18.18) | <0.001 |  |
| Female | 7.41 (2.18-25.17) | 0.002 |  |
| **Change rate of WC** |  |  | 0.859 |
| Male | 2.63 (1.76-3.91) | <0.001 |  |
| Female | 2.86 (1.69-4.84) | <0.001 |  |
| **Change rate of WHtR**^†^ |  |  | 0.688 |
| Male | 4.84 (2.52-9.33) | <0.001 |  |
| Female | 4.54 (2.05-10.08) | 0.002 |  |
| **Age (Phase 7), yrs** | | | |
| **Change rate of BMI** |  |  | 0.277 |
| < 60 | 7.80 (2.52-24.16) | <0.001 |  |
| ≥60 | 5.58 (1.97-15.79) | 0.002 |  |
| **Change rate of WC** |  |  | 0.514 |
| < 60 | 2.80 (1.78-4.43) | <0.001 |  |
| ≥60 | 2.51 (1.64-3.84) | <0.001 |  |
| **Change rate of WHtR**^†^ |  |  | 0.290 |
| < 60 | 5.77 (2.76-12.07) | <0.001 |  |
| ≥60 | 4.15 (2.12-8.12) | <0.001 |  |

^†^ Hazard ratio (Per 0.01 Increase per year) for diabetes;

HR, hazard ratio; CI, confidence interval; BMI, body mass index; WC, waist circumference; WHtR, waist-to-height ratio;

Sex stratification: controlling covariates for initial indicator value, age, ethnicity, family history of diabetes, education level, and covariates from both Phase 3 and Phase 7, including smoking, drinking, socioeconomic position, physical activity, and dietary behavior; for females, additionally controlling menopause status (Phase 3 and Phase 7);

Age stratification: controlling covariates for initial indicator value, sex, ethnicity, family history of diabetes, education level, and covariates from both Phase 3 and Phase 7, including smoking, drinking, socioeconomic position, physical activity, and dietary behavior**.**

**Table S10 Comparison between persons with incident diabetes between Phase 3 and Phase 7 and included participants (using raw adiposity values)**

| **Variable** | **Persons with incident diabetes between Phase 3 and Phase 7 (n = 476)** | **Study population**  **(n = 5666)** | **P value**^†^ |
| --- | --- | --- | --- |
| **Age at Phase 7,** mean±SD, **yrs** | 62.85 ± 6.17 | 61.15 ± 8.03 | <0.001 |
| ≥ 60, N (%) | 303/476 (63.7) | 2850/5666 (50.3) | <0.001 |
| **Sex (Female), N (%)** | 165/476 (34.7) | 1643/5666 (29.0) | 0.011 |
| **Ethnicity (non-white), N (%)** | 88/476 (18.5) | 379/5660 (6.7) | <0.001 |
| **BMI at Phase 3,** mean±SD, **kg/m^2^** | 27.58 ± 4.82 | 24.99 ± 3.45 | <0.001 |
| ≥25, N (%) | 306/453 (67.5) | 2457/5490 (44.8) | <0.001 |
| **BMI at Phase 7,** mean±SD, **kg/m^2^** | 29.07 ± 5.40 | 26.53 ± 4.18 | <0.001 |
| **Slope of BMI from Phase 3 to Phase 7,** mean±SD, **kg/m^2^ per year** | 0.13 ± 0.09 | 0.14 ± 0.11 | 0.462 |
| **WC at Phase 3,** mean±SD, **cm** | 89.13 ± 12.93 | 83.07 ± 11.08 | <0.001 |
| ≥cut-off,^‡^ N (%) | 257/452 (56.9) | 1704/5447 (31.3) | <0.001 |
| **WC at Phase 7,** mean±SD, **cm** | 95.74 ± 12.03 | 90.72 ± 12.05 | <0.001 |
| **Slope of WC from Phase 3 to Phase 7,** mean±SD, **cm per year** | 0.66 ± 0.19 | 0.68 ± 0.25 | 0.126 |
| **WHtR at Phase 3,** mean±SD | 0.53 ± 0.07 | 0.48 ± 0.06 | <0.001 |
| ≥0.5, N (%) | 293/452 (64.8) | 1951/5440 (35.9) | <0.001 |
| **WHtR at Phase 7,** mean±SD | 0.57 ± 0.08 | 0.53 ± 0.07 | <0.001 |
| **Slope of WHtR from Phase 3 to Phase 7,** mean±SD, **** 100* per year** | 0.44 ± 0.11 | 0.43 ± 0.16 | 0.383 |
| **Family history of Diabetes, N (%)** | 105/462 (22.7) | 549/5585 (9.8) | <0.001 |
| **Education level** |  |  | 0.632 |
| Low | 18/359 (5.0) | 192/4190 (4.6) |  |
| Middle | 187/359 (52.1) | 2093/4190 (50.0) |  |
| High | 154/359 (42.9) | 1905/4190 (45.5) |  |
| **Covariate at Phase 3** |  |  |  |
| **Smoking status, N (%)** |  |  | 0.005 |
| Never smoker | 192/429 (44.8) | 2580/5240 (49.2) |  |
| Ex-smoker | 164/429 (38.2) | 2045/5240 (39.0) |  |
| Current smoker | 73/429 (17.0) | 615/5240 (11.7) |  |
| **Drinking status, N (%)** |  |  | 0.001 |
| Non-current | 104/450 (23.1) | 907/5521 (16.4) |  |
| Current moderate | 246/450 (54.7) | 3221/5521 (58.3) |  |
| Current heavy | 100/450 (22.2) | 1393/5521 (25.2) |  |
| **Fruit/vegetable consumption, N (%)** |  |  | <0.001 |
| < Daily | 204/451 (45.2) | 1986/5519 (36.0) |  |
| ≥ Daily | 247/451 (54.8) | 3533/5519 (64.0) |  |
| **Physical activity** |  |  | 0.001 |
| Inactive | 112/451 (24.8) | 999/5525 (18.1) |  |
| Moderate | 162/451 (35.9) | 2011/5525 (36.4) |  |
| Active | 177/451 (39.2) | 2515/5525 (45.5) |  |
| **Socioeconomic position, N (%)** |  |  | <0.001 |
| Low | 99/450 (22.0) | 669/5522 (12.1) |  |
| Intermediate | 131/450 (29.1) | 2342/5522 (42.4) |  |
| High | 220/450 (48.9) | 2511/5522 (45.5) |  |
| **Covariate at Phase 7** |  |  |  |
| **Smoking status, N (%)** |  |  | 0.073 |
| Never smoker | 205/469 (43.7) | 2763/5616 (49.2) |  |
| Ex-smoker | 223/469 (47.5) | 2414/5616 (43.0) |  |
| Current smoker | 41/469 (8.7) | 439/5616 (7.8) |  |
| **Drinking status, N (%)** |  |  | <0.001 |
| Non-current | 126/464 (27.2) | 879/5553 (15.8) |  |
| Current moderate | 226/464 (48.7) | 2924/5553 (52.7) |  |
| Current heavy | 112/464 (24.1) | 1750/5553 (31.5) |  |
| **Fruit/vegetable consumption, N (%)** |  |  | 0.843 |
| < Daily | 118/468 (25.2) | 1376/5574 (24.7) |  |
| ≥ Daily | 350/468 (74.8) | 4198/5574 (75.3) |  |
| **Physical activity** |  |  | <0.001 |
| Inactive | 55/465 (11.8) | 380/5548 (6.8) |  |
| Moderate | 70/465 (15.1) | 629/5548 (11.3) |  |
| Active | 340/465 (73.1) | 4539/5548 (81.8) |  |
| **Socioeconomic position, N (%)** |  |  | <0.001 |
| Low | 92/455 (20.2) | 630/5565 (11.3) |  |
| Intermediate | 145/455 (31.9) | 2513/5565 (45.2) |  |
| High | 218/455 (47.9) | 2422/5565 (43.5) |  |

^†^ T-test or Wilcoxon rank-sum test for continuous variables and chi-square test for categorical variables;

^‡^ Cut-point for male is 90 cm, for female is 80 cm;

BMI, body mass index; WC, waist circumference; WHtR, waist-to-height ratio; Drinking: current moderate (1-14 unit/w), current heavy (>14 unit/w); Socioeconomic position: defined by either current or last recorded employment grade; Physical activity: inactive (<1 hr/week of moderate physical activity and <1 hr/week of vigorous physical activity), active (>2.5 hrs/week of moderate physical activity or >1 hr/week of vigorous physical activity), or moderately active (if not active or inactive).

**Figure S1 Directed acyclic graph for the study variables**


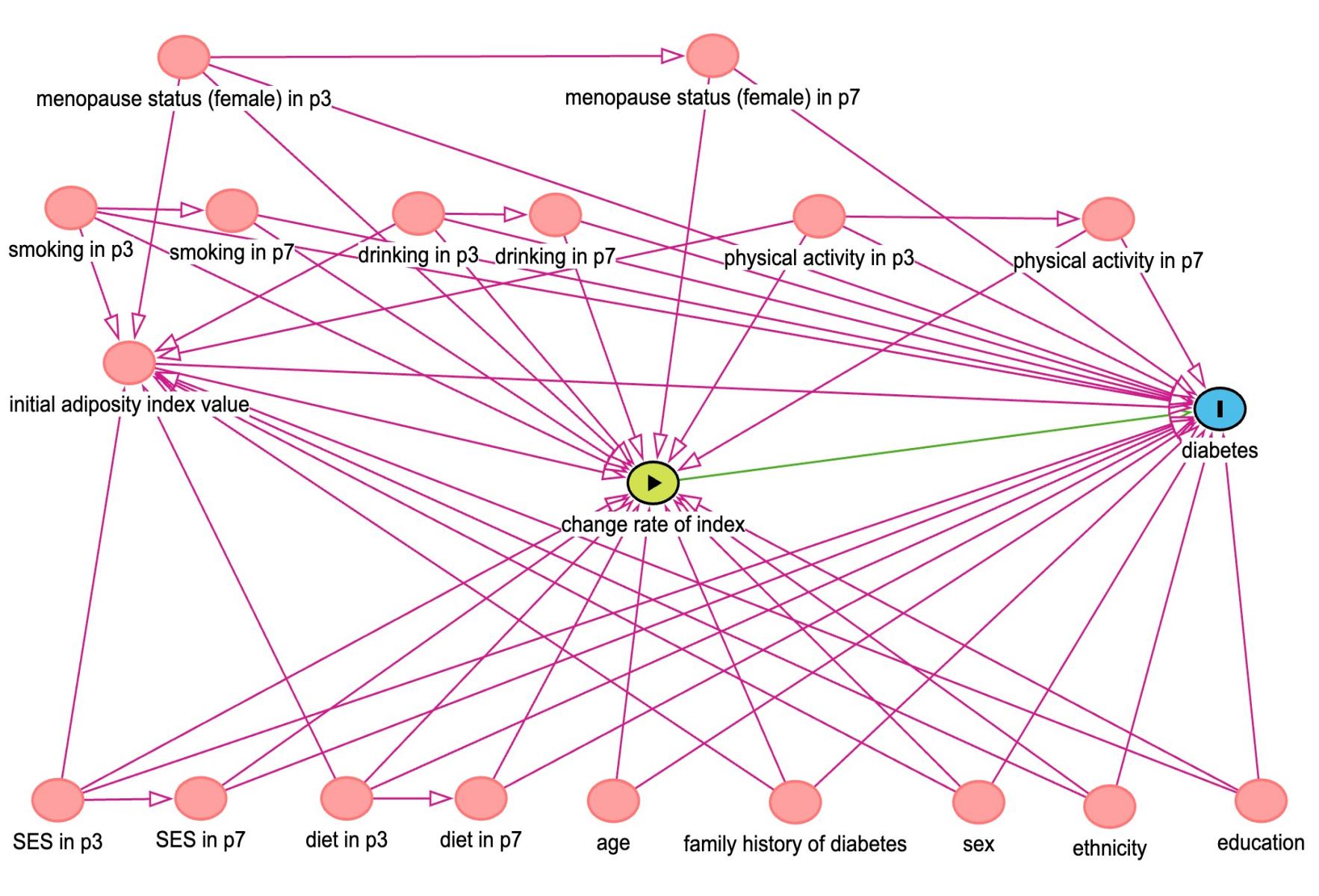


SES, socioeconomic position; p, Phase

**Figure S2 Unconditional estimated trajectories of average BMI, WC, and WHtR (95% CI) from Phase 3 (year 0) to Phase 7**

**a. BMI**


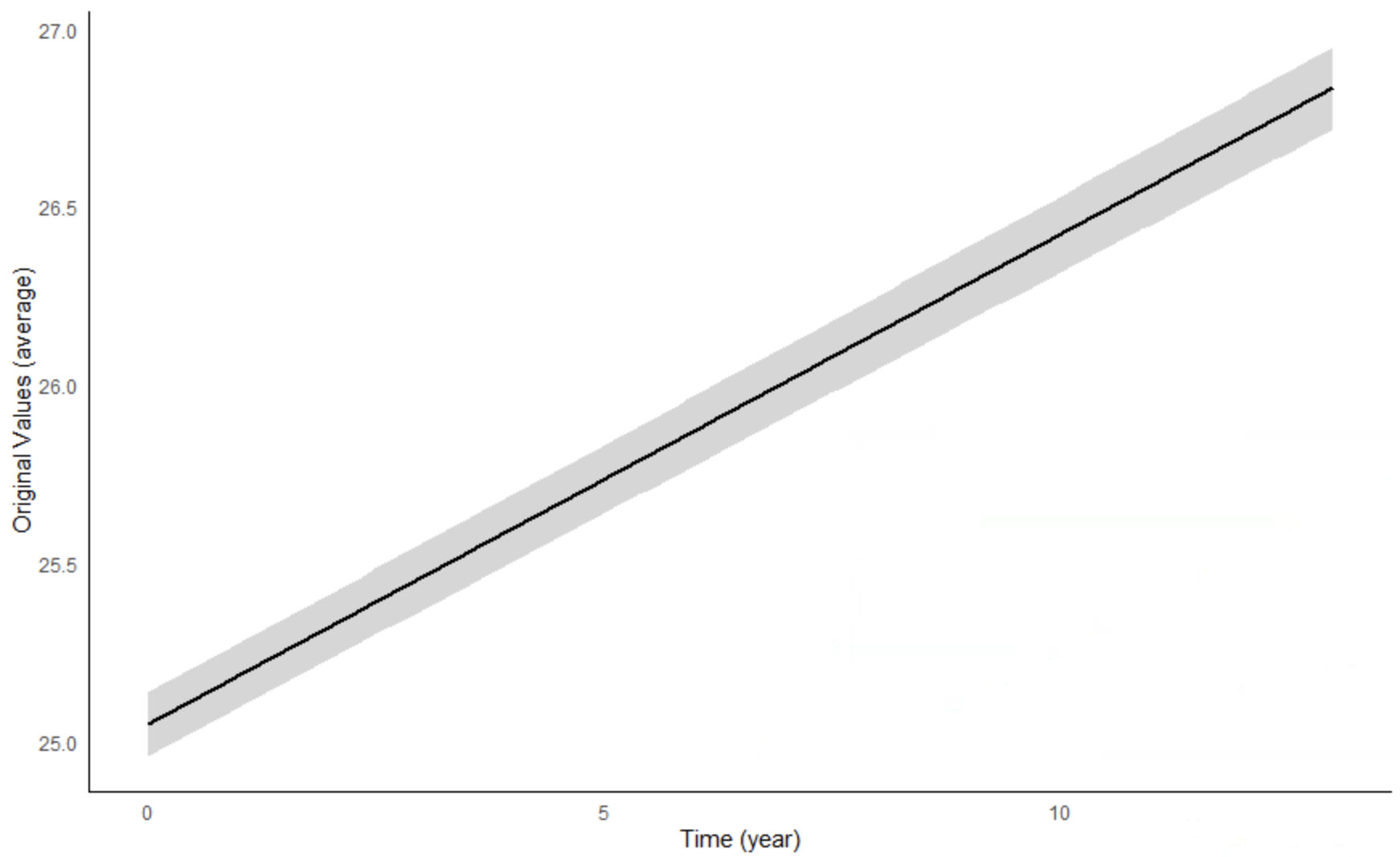


**b. WC**


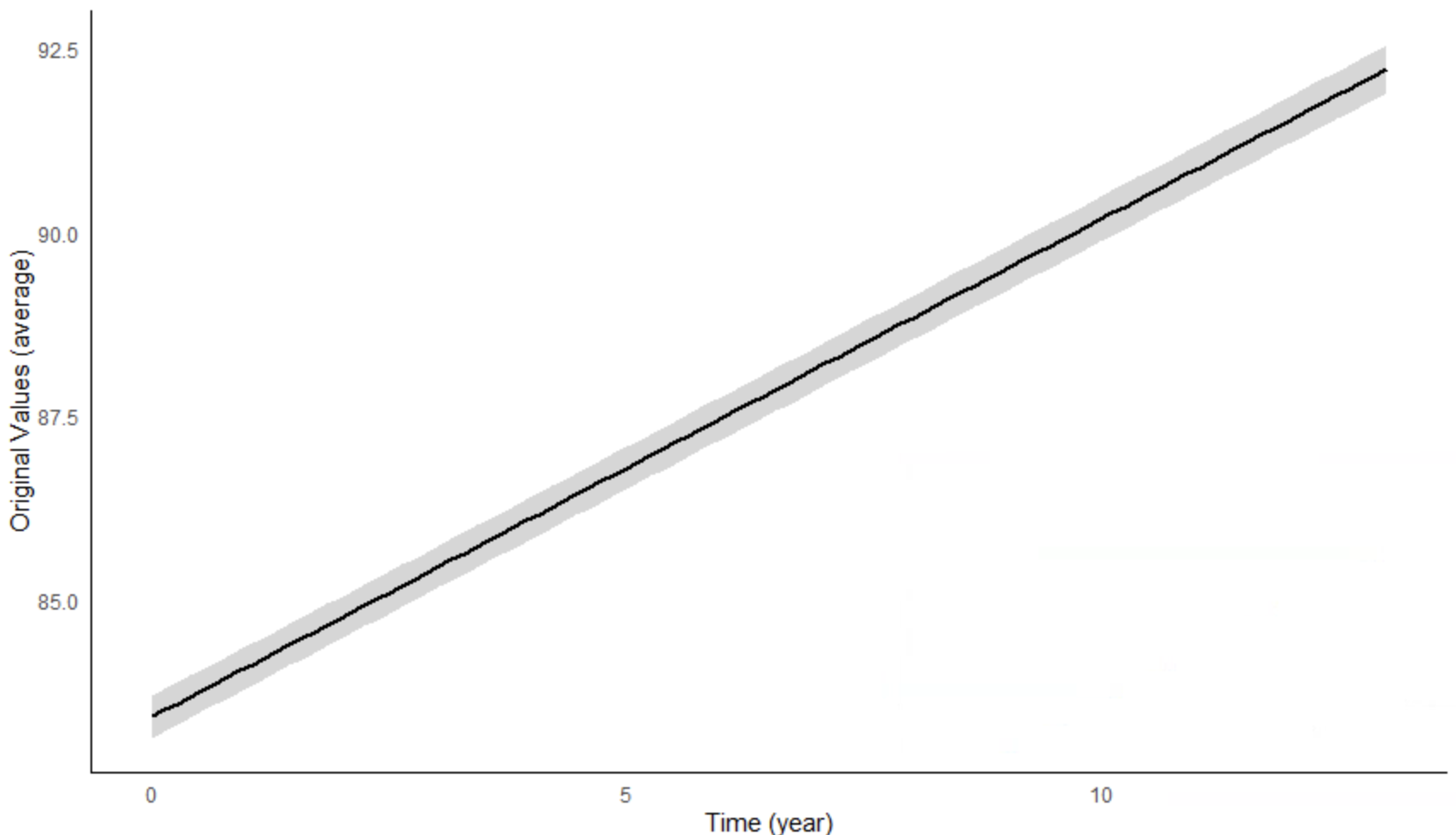


**c. WHtR**


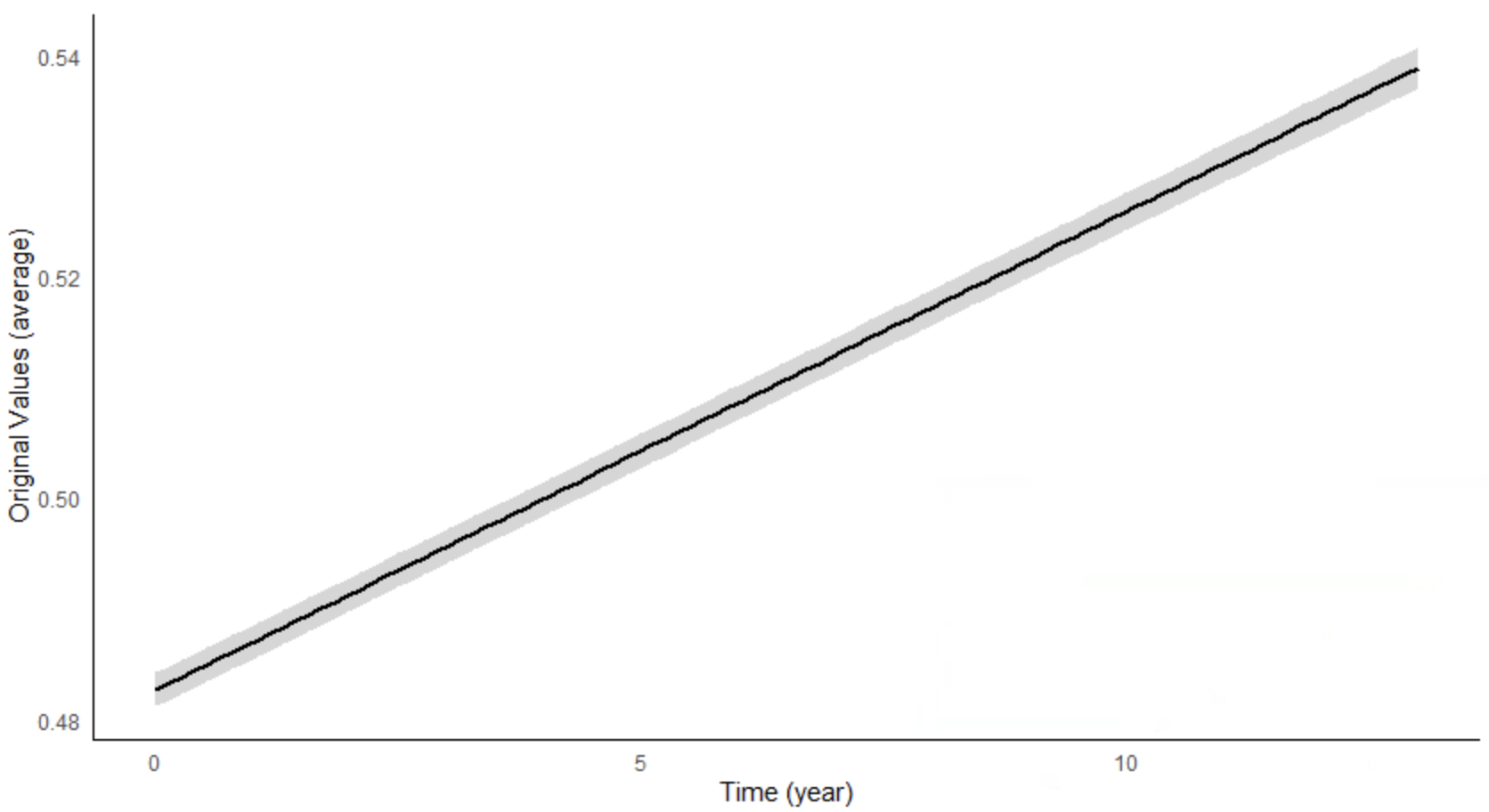


BMI, body mass index; WC, waist circumference; WHtR, waist-to-height ratio; CI, confidence interval;

Time: time after Phase 3 (year 0).
